# Supplementary material for: Agreement of Image Quality Metrics with Radiological Evaluation in the Presence of Motion Artifacts
Source: ArXiv. 2024 Dec 24:arXiv:2412.18389v1. Preprint. [Version 1] (PMC11703327)
Supplement: Supplementary file 1 [file NIHPP2412.18389v1-supplement-1.pdf]

Supplementary Material for:
Agreement of Image Quality Metrics with Radiological Evaluation in the Presence of Motion Artifacts

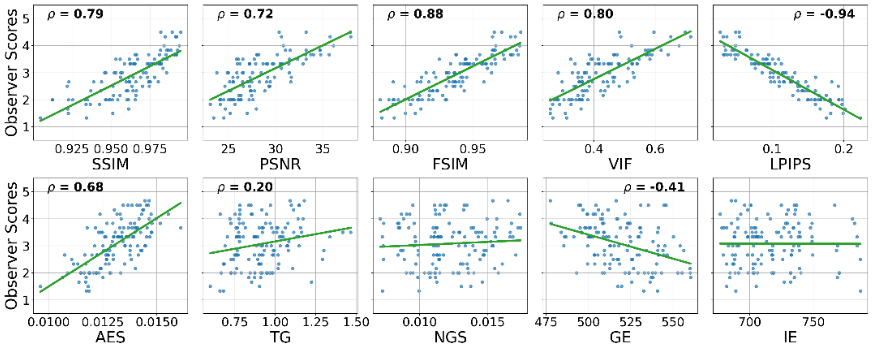

(a) Scatter plot for T_2 FLAIR images.

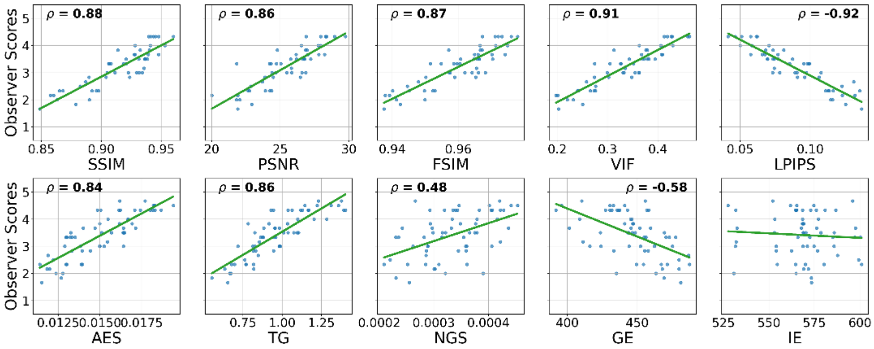

(b) Scatter plot for T_1 TIRM images.

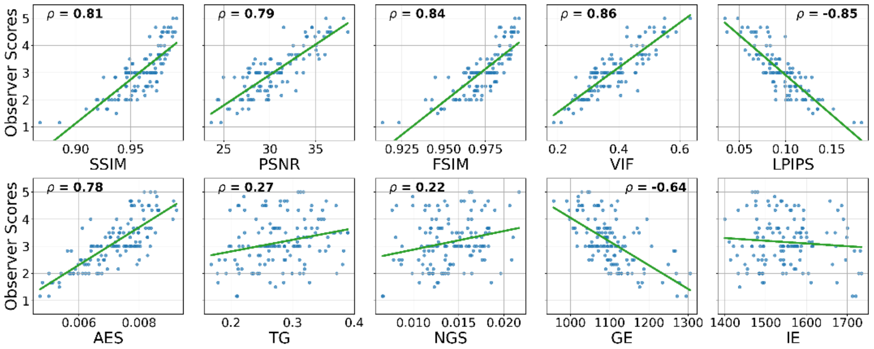

(c) Scatter plot for T_2 TSE images.

FIGURE S1 Scatter plots visualizing the distribution of metrics values against observer scores for the T_2 FLAIR, T_1 TIRM and T_2TSE sequences of the NRU dataset. Each blue dot represents one MPRAGE image volume and the corresponding regression line is shown green. For statistically significant correlations (p-value < 0.05), the corresponding Spearman correlation coefficient is provided on top of the plot. The metrics were calculated with the pre-processing settings {*Multiply, Percentile, Worst*}. Non-integer observer scores result from averaging the scores across the four raters.

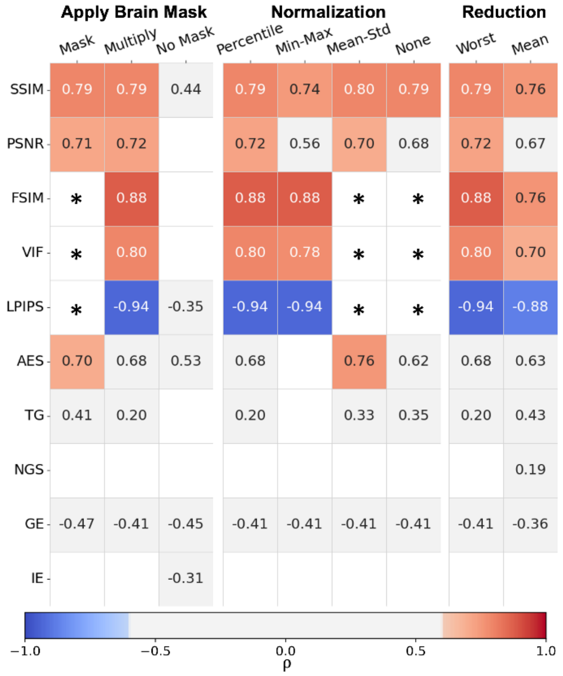

FIGURE S2 Overview on the effect of pre-processing implementations in the correlation between IQM and observers' scores for the T_1 TIRM sequence from the NRU dataset. The table only shows statistically significant correlations ($p < 0.05$), leaving the box empty if this requirement is not fulfilled. We indicated with a "*" values for FSIM, VIF and LPIPS which are not available in case of normalization using "Mean-Std" and "None", as they require a specific range of values (see Table 1). Similarly, these values are unavailable with the "Mask" setting, as the metrics are computed across the entire matrix.

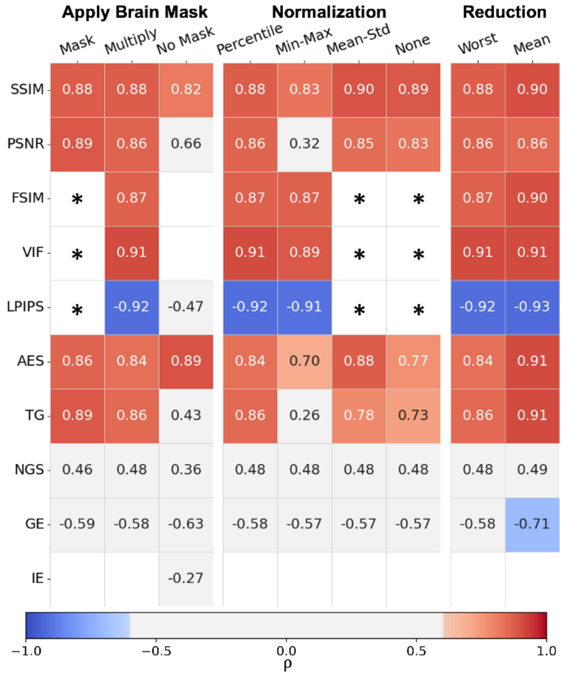

FIGURE S3 Overview on the effect of pre-processing implementations in the correlation between IQM and observers' scores for the T_2 FLAIR sequence from the NRU dataset. The table only shows statistically significant correlations ($p < 0.05$), leaving the box empty if this requirement is not fulfilled. We indicated with a "*" values for FSIM, VIF and LPIPS which are not available in case of normalization using "Mean-Std" and "None", as they require a specific range of values (see Table 1). Similarly, these values are unavailable with the "Mask" setting, as the metrics are computed across the entire matrix.

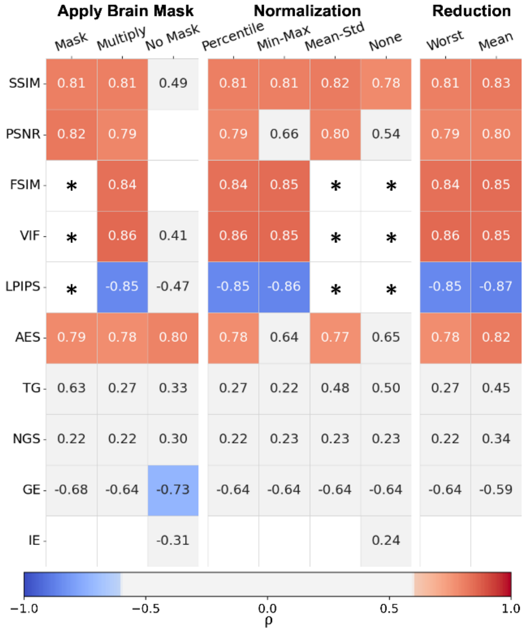

FIGURE S4 Overview on the effect of pre-processing implementations in the correlation between IQM and observers' scores for the T_2 TSE sequence from the NRU dataset. The table only shows statistically significant correlations ($p < 0.05$), leaving the box empty if this requirement is not fulfilled. We indicated with a "*" values for FSIM, VIF and LPIPS which are not available in case of normalization using "Mean-Std" and "None", as they require a specific range of values (see Table 1). Similarly, these values are unavailable with the "Mask" setting, as the metrics are computed across the entire matrix.
